# Regression of Mucosa-Associated Lymphoid Tissue Lymphoma Arising From the Nonampullary Descending Part of the Duodenum by Treatment With Antibiotics in a Helicobacter pylori-Negative Patient

**DOI:** 10.7759/cureus.37194

**Published:** 2023-04-06

**Authors:** Akira Tari, Hideharu Okanobu, Takehiro Tanaka, Tetsuya Tabata, Tadashi Yoshino

**Affiliations:** 1 Department of Gastroenterology, Chugoku Central Hospital, Fukuyama, JPN; 2 Department of Gastroenterology, Takanobashi Central Hospital, Hiroshima, JPN; 3 Department of Gastroenterology, Hiroshima Red Cross Hospital & Atomic-Bomb Survivors Hospital, Hiroshima, JPN; 4 Department of Pathology, Okayama University Graduate School of Medicine, Dentistry and Pharmaceutical Sciences, Okayama, JPN

**Keywords:** nonampullary descending part of the duodenum, microorganism, antibiotic treatment, eradication, helicobacter pylori, mucosa-associated lymphoid tissue lymphoma

## Abstract

We report a 63-year-old male, *Helicobacter pylori*-negative patient with mucosa-associated lymphoid tissue (MALT) lymphoma of the second part of the duodenum that regressed after antibiotic treatment. Esophagogastroduodenoscopy (EGD) showed flat elevation with shallow depression on the contralateral side of the ampulla of Vater. The lesion was limited to the duodenal second part. The patient had a history of *Helicobacter pylori* positivity, with successful eradication at 41 years of age. Twelve months after vonoprazan (VPZ)-based antibiotic treatment, the duodenal lesion had obviously regressed, and the pathological diagnosis was complete histological response (ChR). This case suggests that certain bacteria may promote the development of duodenal MALT lymphoma.

## Introduction

The first-line therapy for mucosa-associated lymphoid tissue (MALT) lymphoma of the stomach has been the eradication of Helicobacter pylori (H. pylori) since the first report by Wotherspoon et al. in 1993 [1]. Although the stomach is the main site for H. pylori colonization, recent reports have described the regression of MALT lymphoma in extragastric areas of the gut after H. pylori eradication, and a pathogenic role of H. pylori in the development of extragastric MALT lymphoma has been emphasized [2-5]. Because of its rarity, little is known about the clinicopathological characteristics and treatment strategy of MALT lymphoma arising from the duodenum. In Japan, duodenal MALT lymphoma was reported to account for only 3.6% of MALT lymphomas of the gastrointestinal tract. In contrast, primary gastric MALT lymphomas make up 77% of cases [6]. This is a case report of an H. pylori-negative patient with MALT lymphoma of the second part of the duodenum that regressed after treatment with antibiotics, suggesting that bacteria other than H. pylori caused the development of MALT lymphoma, which arose from the distal part of the second part of the duodenum.

## Case presentation

A 63-year-old male with no subjective symptoms underwent regular medical checkups. Esophagogastroduodenoscopy (EGD) showed flat elevation in the second part of the duodenum on the contralateral side of the ampulla of Vater. Based on the histopathological findings of biopsy specimens, the patient was diagnosed as having duodenal MALT lymphoma and referred to our hospital for precise examination and treatment. He had a history of ulcers in the duodenal bulb and was diagnosed with *H. pylori* infection, which was successfully eradicated when he was 41 years old. Since then, he underwent yearly EGD examinations for preventive care, but there was no recurrence of the duodenal ulcer, and no remarkable findings were noted.

At the time of evaluation, his superficial lymph nodes were not palpable by physical examination. The levels of serum immunoglobulins (Ig), including IgG, IgM, and IgA, were normal, and no M protein or Bence-Jones proteins were found by immunoelectrophoresis of the serum and urine. The levels of T3, T4, and thyroid-stimulating hormone (TSH) were within the normal range, and all autoimmune disease markers, such as anti-thyroglobulin antibody, antinuclear antibody, rheumatoid factor, and autoantibodies against anti-Sjögren’s syndrome-related antigen A (SSA) and B (SSB), were negative. Examination of the sternal marrow showed no abnormalities. The successful eradication of *H. pylori* was confirmed by negative serum IgG titers for *H. pylori* (4.5<10) (*H. pylori*-LATEX “SEIKEN,” Denka Company Ltd., Niigata, Japan), a negative rapid urease test (Helicocheck; Otsuka Pharmaceutical Co., Ltd. Tokyo, Japan), and a negative urea breath test (0<2.5) (UBIT; Otsuka Pharmaceutical Co., Ltd. Tokyo, Japan).

**Figure 1:**
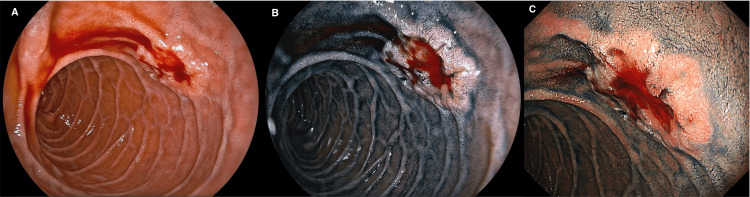
Endoscopic images showing MALT lymphoma in the nonampullary duodenal second part before antibiotic treatment. (A) In white light. (B) In chromoendoscopy using indigo carmine, distant view. (C) In chromoendoscopy using indigo carmine, closer view. MALT: mucosa-associated lymphoid tissue

EGD (Olympus, Tokyo, Japan) showed flat elevation with shallow depression mimicking a 0-IIa+IIc lesion of early gastric cancer with bridging folds on the contralateral side of the ampulla of Vater (Figure 1). Endoscopic insufflation caused oozing from the lesion. There were no abnormal findings in the stomach or in other parts of the duodenum. There were no abnormal findings on contrast-enhanced computed tomography (CT/CE+) scans (General Electric, Fairfield, Connecticut, USA) of the neck, chest, abdomen, and pelvis and ^18^F-fluorodeoxyglucose (FDG) positron emission tomography combined with computed tomography (PET-CT) (General Electric, Fairfield, Connecticut, USA), total colonoscopy, and both video capsule endoscopy (VCE) (Medtronic PLC, Minneapolis, USA) and double-balloon enteroscopy (DBE) (Fujinon, Tokyo, Japan) of the small intestine.

**Figure 2:**
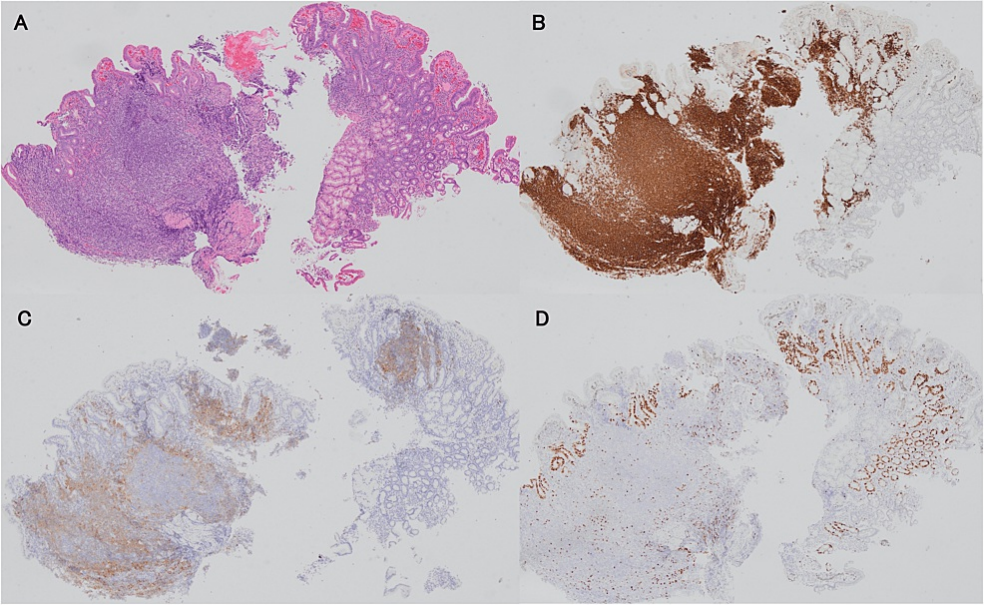
Histological findings of biopsy specimens from the duodenal lesion before treatment. (A) Dense mononuclear cell proliferation in the duodenal mucosa (hematoxylin and eosin staining). Immunohistochemically, tumor cells were positive for CD20 (B) and IRTA-1 (C). The Ki-67 labeling index was low (D). IRTA-1: immune receptor translocation-associated protein-1

The biopsied specimens from the duodenal lesion showed diffuse proliferation of medium-sized cells. Immunohistochemically, the tumor cells were positive for cluster of differentiation (CD) 20 (CD20), B-cell leukemia/lymphoma-2 (BCL-2), and immune receptor translocation-associated protein-1 (IRTA-1) and negative for CD3, CD5, CD10, and BCL-6; the Ki-67 labeling index was low (Figure 2). Molecular cytogenetic studies conducted using fluorescence in situ hybridization (FISH) were negative for the 11;18 translocation, t (11;18) (q21;q21). This lesion was diagnosed as MALT lymphoma without the *BIRC3-MALT1* fusion/translocation according to the histological, immunophenotypic, and molecular findings. This patient was diagnosed with clinical stage I (Lugano staging system) [7], and the lesion existed only in the duodenal second part. The biopsied specimens from the duodenal bulb showed gastric metaplasia, according to positivity for MAC5AC.

Because the involvement of certain bacteria was considered and the patient had been previously infected with *H. pylori*, he was treated with vonoprazan (VPZ)-based triple therapy (VPZ 20 mg + amoxicillin 750 mg + clarithromycin 200 mg) twice a day for seven days, simulating *H. pylori *eradication treatment, followed by a “watch-and-wait” strategy. During the “watch-and-wait” observation period, the patient has observed progress according to the protocol for duodenal-type follicular lymphoma (Table 1) [8].

**Table 1:** Examination during the observation period of the “watch-and-wait” protocol. CT: computed tomography, PET-CT: positron emission tomography combined with computed tomography

Observation period	Examination
Every four months	Symptoms, swelling of the superficial lymph node, blood test, esophagogastroduodenoscopy
Every year	Video capsule endoscopy, colonoscopy, PET-CT, or contrast-enhanced CT scan (neck and pelvis)

**Figure 3:**
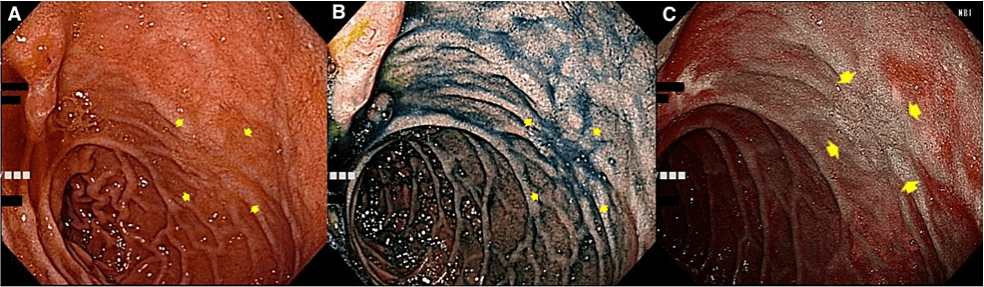
Endoscopic images showing MALT lymphoma in the nonampullary duodenal second part at 12 months after antibiotic treatment. (A) In white light. (B) In chromoendoscopy using indigo carmine, distant view. (C) In narrow-band imaging. MALT: mucosa-associated lymphoid tissue

**Figure 4:**
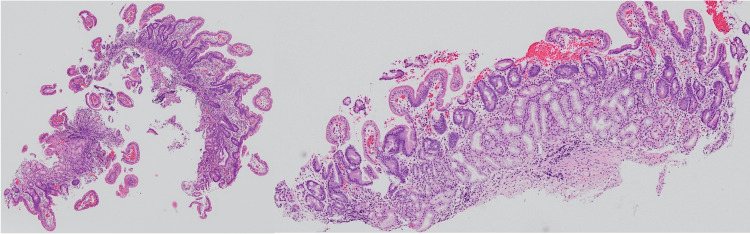
Histological findings of biopsy specimens from the duodenal lesion at 12 months after antibiotic treatment. Hematoxylin and eosin staining, original magnification 16×

EGD performed eight months after eradication therapy showed improvement of the MALT lymphoma lesion to probable minimal residual disease (pMRD) [9,10]. Twelve months after eradication therapy, regression of the duodenal lesion was obvious, and there was only a slightly depressed irregular lesion on the contralateral side of the ampulla of Vater (Figure 3). The pathological diagnosis for the biopsy specimens from the lesion was complete histological response (ChR) (Figure 4). This patient showed no evidence of MALT lymphoma recurrence and maintained clinical complete remission (CR) at 40 months after eradication therapy, according to the protocol for duodenal-type follicular lymphoma.

## Discussion

MALT lymphoma, which arose from the second part of the duodenum in our patient, clearly regressed after treatment with antibiotics. The assumption that a type of microorganism other than *H. pylori* was involved in the development of MALT lymphoma in this case seems reasonable. The possibility can be supported by the fact that Mediterranean lymphoma of the small intestine, a prototype form of MALT lymphoma [11], has pathological and immunological similarities with MALT lymphoma of the stomach and can be treated successfully with antibiotics at an early stage [12], although the causative pathogen has not yet been identified [13]. The present case suggests that *H. pylori* may not be involved in the pathogenesis of extragastric MALT lymphoma, which regressed by antibiotic treatment in an *H. pylori-*positive patient.

It is now widely accepted that *H. pylori* infection is involved in the pathogenesis of MALT lymphoma of the stomach [1]. It is well known that *H. pylori* infects the ectopic or metaplastic gastric mucosa of the duodenal bulb [14,15]. It has been reported that MALT lymphoma arising from the duodenal bulb may be derived from ectopic gastric mucosa or gastric epithelial metaplasia [16,17]. Thus, it has been suggested that *H. pylori* in the stomach plays a role in the development of MALT lymphoma in the duodenal bulb [4]. On the other hand, the clinicopathological characteristics of duodenal MALT lymphomas have been reported to differ between cases arising from the bulb and those arising from the descending to ascending part of the duodenum [18]. In Japan, the infection rates of *H. pylori* did not differ between the two groups, but the therapeutic effects (CR+pMRD) of *H. pylori* eradication alone were less prominent in cases involving the primary descending part of the duodenum (21%) than in those involving the primary bulb of the duodenum (57.1%). These findings suggest that pathogenesis and pathophysiology differences exist in cases involving the duodenal bulb and the descending part. Autoimmune mechanisms have been suggested to be involved in the etiology of primary duodenal MALT lymphoma [19,20].

## Conclusions

Our *H. pylori*-negative patient with MALT lymphoma of the second part of the duodenum regressed after antibiotic treatment. Because MALT lymphoma in our patient developed in the distal part of the duodenum in the absence of gastric metaplasia and without *H. pylori* infection or autoimmune abnormality, this case suggests that certain bacteria may somehow be responsible for the development of the disease.

Further studies with more cases are necessary to determine the pathogenesis and pathophysiology of MALT lymphoma arising from the descending to ascending part of the duodenum.
